# Temporal patterns of inflammation-related proteins measured in the cerebrospinal fluid of patients with aneurysmal subarachnoid hemorrhage using multiplex Proximity Extension Assay technology

**DOI:** 10.1371/journal.pone.0263460

**Published:** 2022-03-24

**Authors:** Pavlos Vlachogiannis, Lars Hillered, Per Enblad, Elisabeth Ronne-Engström

**Affiliations:** Department of Neurosciences, Neurosurgery, Uppsala University, Uppsala, Sweden; Barrow Neurological Institute, UNITED STATES

## Abstract

**Background:**

The complexity of the inflammatory response post subarachnoid hemorrhage (SAH) may require temporal analysis of multiple protein biomarkers simultaneously to be more accurately described.

**Methods:**

Ventricular cerebrospinal fluid was collected at days 1, 4 and 10 after SAH in 29 patients. Levels of 92 inflammation-related proteins were simultaneously measured using Target 96 Inflammation ^®^ assay (Olink Proteomics, Uppsala, Sweden) based on Proximity Extension Assay (PEA) technology. Twenty-eight proteins were excluded from further analysis due to lack of >50% of measurable values. Temporal patterns of the remaining 64 proteins were analyzed. Repeated measures ANOVA and its nonparametric equivalent Friedman’s ANOVA were used for comparisons of means between time points.

**Results:**

Four different patterns (Groups A-D) were visually observed with an early peak and gradually decreasing trend (11 proteins), a middle peak (10 proteins), a late peak after a gradually increasing trend (30 proteins) and no specific pattern (13 proteins). Statistically significant early peaks defined as Day 1 > Day 4 values were noticed in 4 proteins; no significant decreasing trends defined as Day 1 > Day 4 > Day 10 values were observed. Two proteins showed significant middle peaks (i.e. Day 1 < Day 4 > Day 10 values). Statistically significant late peaks (i.e. Day 4 < Day 10 values) and increasing trends (i.e. Day 1 < Day 4 < Day 10 values) were observed in 14 and 10 proteins, respectively. Four of Group D proteins showed biphasic peaks and the rest showed stable levels during the observation period.

**Conclusion:**

The comprehensive data set provided in this explorative study may act as an illustration of an inflammatory profile of the acute phase of SAH showing groups of potential protein biomarkers with similar temporal patterns of activation, thus facilitating further research on their role in the pathophysiology of the disease.

## Introduction

Spontaneous subarachnoid hemorrhage (SAH) comprises approximately 5% of all strokes with an incidence of around 9 cases per 100.000 per year and aneurysm rupture being the cause in 85% of cases [1]. Brain injury in SAH occurs both at the time of the bleeding itself, termed primary injury, but also during the following days to weeks, a phenomenon known as secondary injury. Despite advances in the critical care of SAH patients and aneurysm treatment methods, mortality and morbidity rates among the survivals from the initial bleeding remain high, reflecting a lack of effective treatments targeting the pathophysiological mechanisms that underlie the secondary injury.

Delayed cerebral ischemia (DCI) develops in approximately 30–40% of SAH patients and is considered to be a major cause of unfavorable outcome [2]. For decades the condition was attributed to cerebral vasospasm (CV), i.e. the narrowing of basal cerebral arteries seen early on angiography and persisting for up to two weeks post SAH leading to decreased cerebral blood flow (CBF) and infarctions in the affected territories [3]. However, about 20% of SAH patients develop DCI without radiological evidence of CV and only 30% of patients with CV on angiography actually suffer from DCI [4]. Moreover, the randomized multicenter CONCSIOUS trials with the endothelin receptor antagonist Clazosentan failed to show any effect on functional outcome or incidence of cerebral ischemia despite significant decrease of CV [5, 6]. Thus, an uncoupling of angiographic vasospasm and DCI became apparent and new concepts emerged as potential underlying mechanisms of delayed brain injury [7, 8].

Early brain injury (EBI) refers to the events occurring within the first 72h from ictus and includes the primary injury and its direct consequences. Elevation of intracranial pressure (ICP), global ischemia and impairment of CBF autoregulation, cortical spreading depolarization (CSD), disruption of blood—brain barrier (BBB), cell death, oxidative stress and inflammatory processes are among the mechanisms that are activated shortly after aneurysm rupture and evolve during the following days [9].

The processes triggered by the EBI are now believed to play an important role in the development of DCI [9, 10]. Neuroinflammation may be a mechanistic link between these two conditions and has been studied extensively in the past years [11–13]. Many clinical and experimental studies report levels of single or few inflammatory biomarkers (such as cytokines) in the peripheral blood, cerebrospinal fluid (CSF) and cerebral extracellular fluid through microdialysis (MD) in SAH patients or animal models and further correlate these biomarkers with clinical parameters and outcome [14–22]. Although this strategy provides useful insight into the interplay between inflammation and the disease course it fails to account for the complexity of the involved mechanisms that cannot be adequately described by a single (or few) biomarker(s) alone.

The aim of the present explorative study was to provide a comprehensive inflammatory profile of the acute phase of SAH by simultaneously measuring the levels of 92 inflammation-related proteins in the CSF at days 1, 4 and 10 after admission. This was achieved using Target 96 Inflammation^®^ assay (Olink Proteomics, Uppsala, Sweden) based on Proximity Extension Assay (PEA) technology. PEA technology enables measurements of multiple proteins simultaneously in the same samples using only 1 μL of CSF (or other biological sample) with high specificity [23]. The analyzed proteins were then categorized into different groups according to their temporal expression and their peaks and trends throughout the observation period were noted.

## Materials and methods

### Ethics

The study was conducted in accordance with Declaration of Helsinki for human studies and approved by Uppsala University Ethics Committee. All participants or their next of kin gave written consent for participation in the study.

### CSF samples and analysis

Inclusion period was between May 2013 and August 2014. Eligibility criteria was spontaneous SAH severe enough to require insertion of an external ventricular drain (EVD) within 24 h from ictus. Patients considered terminally ill from the bleeding were excluded. Ventricular CSF samples were collected through the EVD within 24 h, on day 4 and between days 9–11 after the bleeding, centrifuged directly after collection and frozen in -70°C. The samples were then analyzed using Target 96 Inflammation ^®^, a multiplex assay panel manufactured by Olink Proteomics AB, Uppsala, Sweden where 92 inflammation-related protein biomarkers are simultaneously measured using Proximity Extension Assay (PEA) technology. The analytical method was previously described in detail [23, 24]. More information can also be found online at https://www.olink.com/. In short, a matched pair of antibodies labeled with unique oligonucleotide tags (“probes”) bind specifically to each target protein present in the sample. The two probes thus come in proximity and can hybridize by enzymatic DNA polymerization producing a DNA sequence that is unique for each protein. Real-time quantification PCR (qPCR) is then used to detect, amplify and quantify these DNA sequences. The number of qPCR cycles required for detection is related to the concentration of the protein in the sample. Data is then digitally processed and relative concentrations of the proteins in each sample are reported. Values are provided in output unit Normalized Protein Expression (NPX) on log2 scale. NPX values express relative quantification between samples but is not an absolute quantification. Limit of detection (LOD) is determined for each biomarker based on the negative controls analyzed in each run.

### Proteins

A list of the 92 inflammation-related proteins included in the panel as well as their families is provided in S1 Data. In summary, the panel included 31 cytokines, 20 chemokines, 9 growth factors, 8 tumor necrosis factor (TNF)-family members, 6 membrane glycoproteins, 6 neurotrophic factors, 5 proteases and 7 miscellaneous proteins.

A total of 87 NPX values (29 patients x 3 time points) corresponding to protein concentrations were collected for each protein. Variations in the detectability of the proteins in the samples were noticed; for example, in 44 proteins all samples were successfully analyzed while in 6 proteins all values were below limit of detection. Inclusion criteria were therefore defined that the values needed to be above level of detection (LOD) in at least 50% of the samples for each protein (that is ≥44 existing values) and in 1/3 of the patients for each day (≥10), which lead to the exclusion of 28 proteins, thus leaving 64 proteins for further analysis.

### Statistics

The temporal pattern of each protein was illustrated in graphs with means and 0.95 confidence intervals. Repeated measures Analysis of variance (ANOVA) with Fisher LSD test for post-hoc analyses was used for comparisons of mean NPX values between time points in those biomarkers where the values were normally distributed (tested with histograms and Shapiro-Wilk’s test) and the assumption of compound symmetry was met (tested with Mauchly’s sphericity test). The nonparametric equivalent Friedman’s ANOVA was used for analysis in biomarkers whose values were not normally distributed and/or the assumption of compound symmetry could not be met (i.e. sphericity test with a p-value < 0.05). When Friedman’s ANOVA indicated significant variance in the mean NPX values in the latter group, further pairwise comparisons were performed with Wilcoxon test to identify between which days the significant differences were found. Missing data were left as such. All results were considered statistically significant at the p<0.05 level. All statistical analyses and graphical presentations were performed using the Statistica^®^ software (version 13.05.0.17, TIBCO Software Inc, Tulsa, OK, USA).

## Results

Demographic data of the 29 patients included in the study are presented in Table 1. Brain CT scans were used to establish the diagnosis of SAH and were classified according to Fisher scale (median 4) [25]. World Federation of Neurosurgical societies (WFNS) scores were noted on admission (median 4) [26]. CT angiography and catheter angiography were performed in all cases and aneurysms were identified in 28 patients. In 26 patients the aneurysms were endovascularly secured and the remaining two were surgically clipped. The patients were treated at the Neurointensive Care Unit of Uppsala University Hospital for at least 10 days after the bleeding. Standardized treatment protocols described in a previous publication were applied [27]. Functional outcome was assessed by a research nurse at 1 year using Glasgow Outcome Scale [28].

**Table 1:** Demographic data of the patient cohort.

Number of patients	29
Male vs Female	12 vs 17
Mean age (range)	57 (37–81)
Median WFNS score	4
Median Fisher grade	4
Anterior vs posterior circulation aneurysm	23 vs 5 (1 patient with no aneurysm)
Embolization vs surgery	26 vs 2
Favorable vs unfavorable outcome (GOS)	9 vs 20

**Fig 1:**
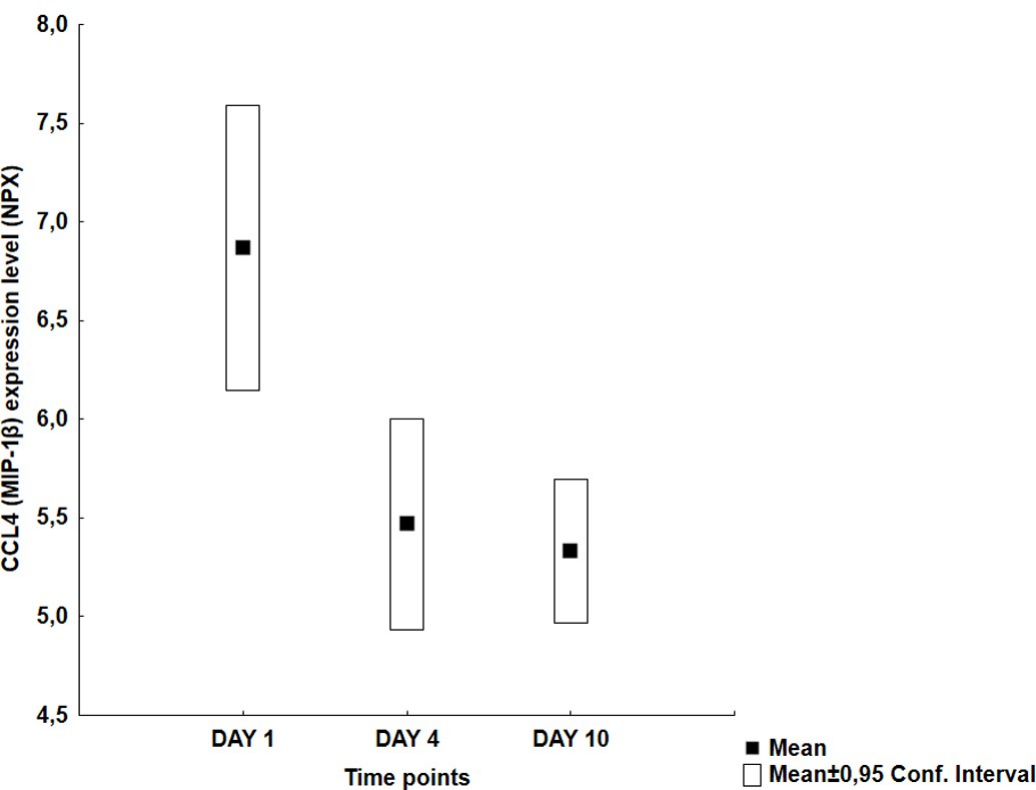
Group A example; CCL4 (MIP-1ß): Temporal pattern of protein expression level (mean NPX ± 0.95 CI) for C-C motif chemokine ligand 4 (CCL4) a.k.a. macrophage inflammatory protein-1ß (MIP-1ß). Statistically significant early peak (mean day 1 vs day 4 values = 6,86 vs 5,46; p<0,005) but no significantly decreasing trend since values seemed to stabilize between day 4 and 10 (5,46 vs 5,33; p = 0,52).

**Fig 2:**
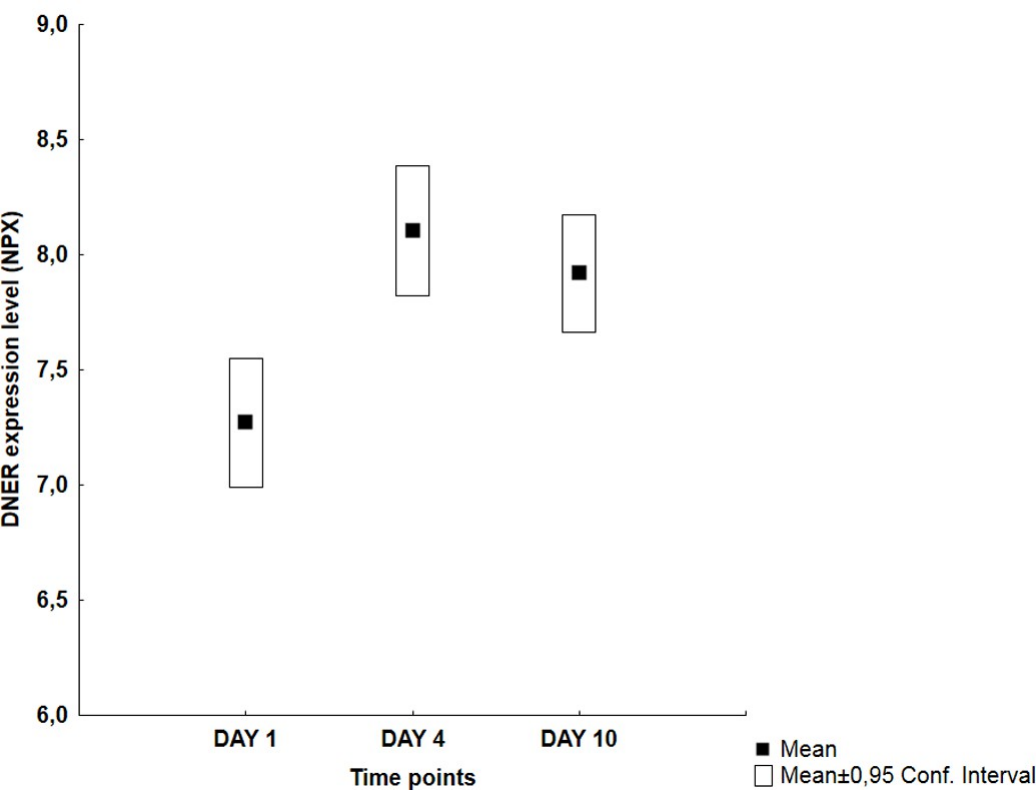
Group B example; DNER: Temporal pattern of protein expression level (mean NPX ± 0.95 CI) for Delta and Notch‐like epidermal growth factor‐related receptor (DNER). Statistically significant peak in the middle of the observation period defined by significantly higher day 4 vs day 1 (8,1 vs 7,27; p<0.005) *and* day 4 vs day 10 mean NPX values (8,1 vs 7,92; p<0.05).

**Fig 3:**
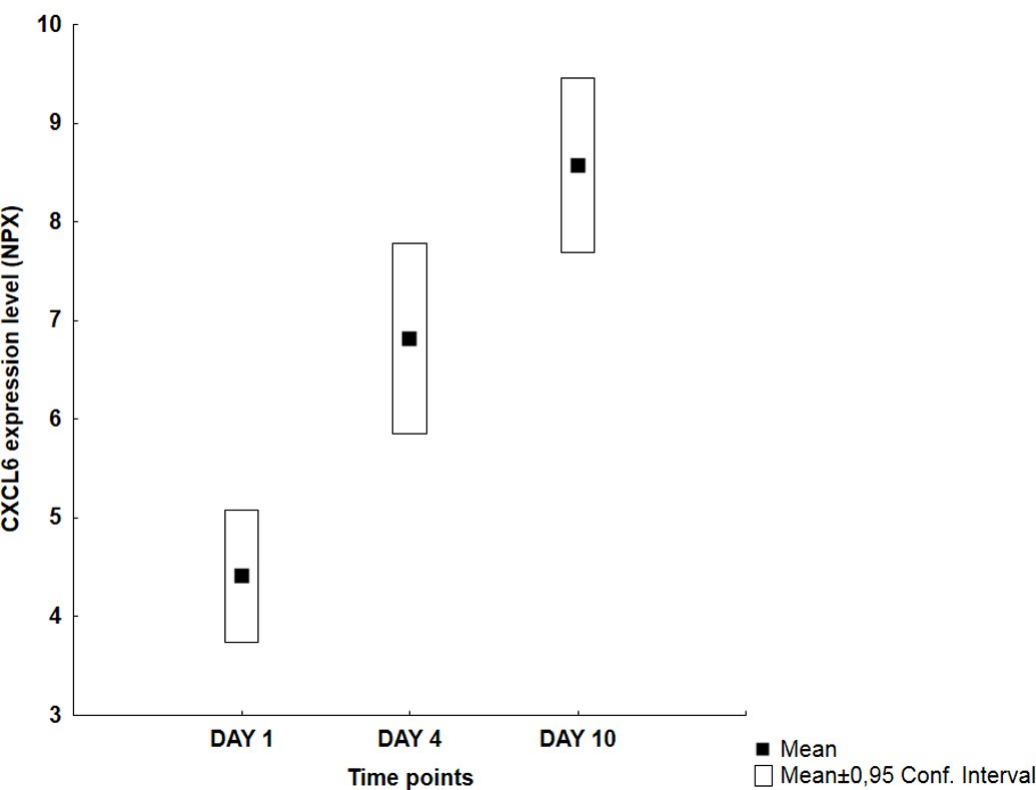
Group C example; CXCL6: Temporal pattern of protein expression level (mean NPX ± 0.95 CI) for C-X-C motif chemokine ligand 6 (CXCL6) showing statistically significant late peak and increasing trend, that is day 1 < day 4 < day 10 mean NPX values (4,41 vs 6,81 vs 8,57 respectively; p<0,005).

**Fig 4:**
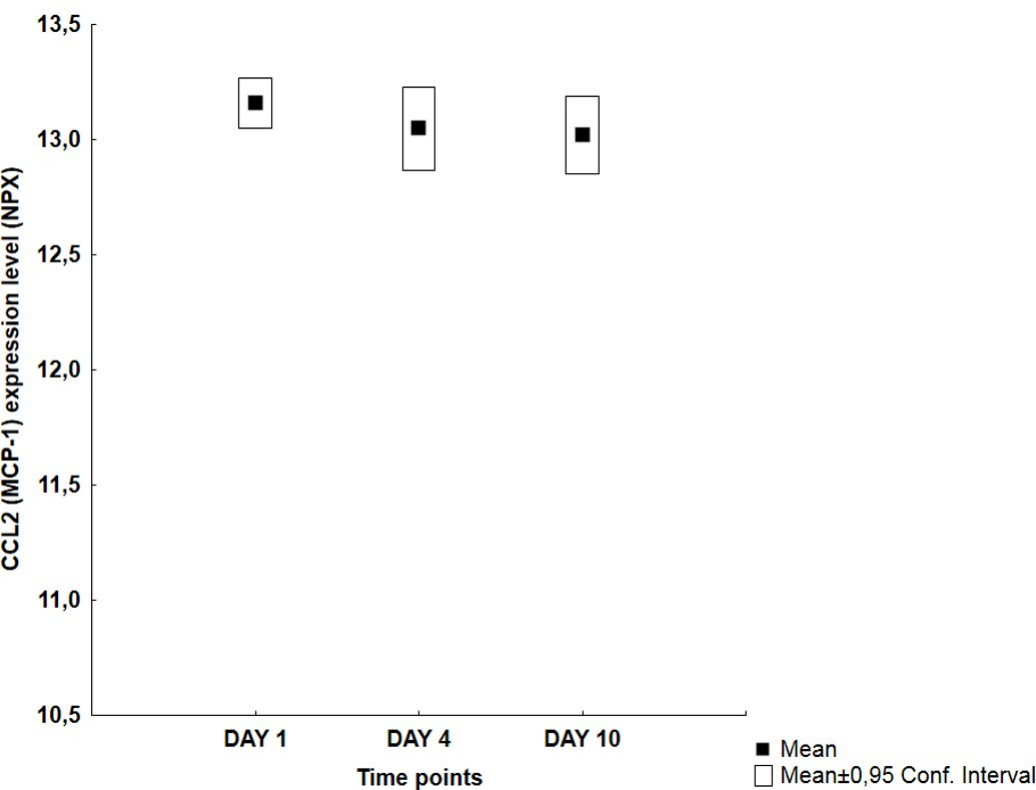
Group D example; CCL2 (MCP-1): Temporal pattern of protein expression level (mean NPX ± 0.95 CI) for C-C motif chemokine ligand 2 (CCL2) a.k.a. monocyte chemotactic protein 1 (MCP-1) showing essentially stable values throughout the whole observation period (13,15 vs 13,04 vs 13,02; p = 0,575).

Visual inspection of the graphs revealed four different temporal patterns (Table 2): an early peak at day 1 followed by a decreasing trend (Group A: 11 proteins), a middle peak at day 4 (Group B: 10 proteins), an increasing trend with a late peak at day 10 (Group C: 30 proteins) and finally no specific pattern (Group D: 13 proteins). Figs 1–4 illustrate typical graphs of protein expression levels (mean NPX and 0.95 confidence intervals) over time for each group.

**Table 2:** Groups of proteins by their temporal pattern of expression.

GROUP A (n = 11)	GROUP B (n = 10)	GROUP C (n = 30)	GROUP D (n = 13)
EARLY PEAK	MIDDLE PEAK	LATE PEAK	NO PATTERN
CCL11	BDNF	4E-BP1	CCL2 (MCP-1)
CCL20	beta-NGF	ADA	CCL25
CCL3 (MIP-1α)	CCL28	CASP-8	CD6
CCL4 (MIP-1β)	CST-5	CCL13 (MCP-4)	CSF-1
CD244	DNER	CCL19	CX3CL1
FGF-19	FGF-5	CCL23 (MIP-3)	CXCL5
FGF-21	Flt3L	CCL7 (MCP-3)	IL-10
LIF	IL-18R1	CCL8 (MCP-2)	IL-12B
MMP10	LIF-R	CD40	MMP1
TGF-α	OSM	CD5	SCF
TRAIL (TNFSF10)		CDCP-1	TNFRSF9
		CXCL1	TNFSF14
		CXCL10	VEGF
		CXCL11	
		CXCL6	
		CXCL8 (IL-8)	
		CXCL9	
		EN-RAGE	
		HGF	
		IL-10RB	
		IL-18	
		IL-6	
		IL-7	
		OPG	
		SIRT-2	
		STAMPB	
		TGF-β1	
		TNF-B	
		TWEAK	
		uPA	

Four Group A proteins (Table 3) showed statistically significant early peak, defined as significantly higher day 1 vs day 4 values, i.e. CCL11, CCL4 (MIP-1β), LIF and TGF-α; no proteins in this group showed statistically significant decreasing trend (that is, significant differences between day 1 vs day 4 *and* day 4 vs day 10 values).

**Table 3:** Group A proteins.

	DAY 1	1 v 4	DAY 4	4 v 10	DAY 10	1 v 10
**CCL11**	3,21 (2,75–3,67)	**	2,36 (2,15–2,58)		2,47 (2,26–2,68)	**
**CCL20**	6,21 (5,25–7,17)		5,97 (5,19–6,75)		5,58 (4,90–6,26)	
**CCL3 (MIP-1)**	3,83 (3,14–4,52)		3,21 (2,69–3,73)		3,19 (2,81–3,56)	
**CCL4 (MIP-1β)**	6,86 (6,14–7,59)	**	5,46 (4,93–6)		5,33 (4,96–5,69)	**
**CD244**	2,24 (1,86–2,62)		1,88 (1,61–2,15)		1,72 (1,52–1,92)	
**FGF-19**	4,22 (3,88–4,57)		3,80 (3,47–4,13)		3,69 (3,31–4,06)	
**FGF-21**	1,74 (1,34–2,14)		1,53 (1,23–1,83)	**	1,03 (0,90–1,15)	**
**LIF**	5,80 (5,10–6,50)	**	4,04 (3,35–4,73)		3,94 (3,34–4,54)	**
**MMP10**	4,09 (3,55–4,63)		3,94 (3,27–4,61)		3,54 (3,16–3,93)	
**TGF-α**	3,06 (2,79–3,33)	*	2,81 (2,61–3,02)		2,66 (2,51–2,80)	*
**TRAIL**	3,57 (3,06–4,07)		2,87 (2,45–3,29)		2,80 (2,52–3,09)	

Comparison of protein expression levels (mean NPX ± 0.95 CI within parentheses) between time points for each protein. Repeated measures ANOVA and Fisher LSD test for post-hoc analyses was used for comparisons when data were normally distributed and assumption of compound symmetry was met. Nonparametric equivalents Friedman’s ANOVA and Wilcoxon test were used when parametric statistics were deemed inappropriate; asterisks indicate level of significance for each comparison (* = p<0,05;

** = p<0,005).

Eight Group B proteins (Table 4) showed significantly higher values between day 4 vs day 1, i.e. Beta-NGF, CCL28, CST5, DNER, FGF-5, Flt3L, LIF-R and OSM). Four of these proteins showed decreasing NPX values towards day 10 and the rest remained stable. The only proteins with statistically significant middle peak (that is, significant difference between day 4 vs day 1 *and* day 4 vs day 10 values) were CCL28 and DNER.

**Table 4:** Group B proteins.

	DAY 1	1v4	DAY 4	4v10	DAY 10	1v10
**BDNF**	2,41 (1,78–3,04)		3,32 (2,35–4,28)		3,37 (2,19–4,54)	
**b-NGF**	0,96 (0,79–1,13)	**	2,08 (1,86–2,31)		1,86 (1,59–2,13)	**
**CCL28**	0,51 (0,38–0,64)	**	0,71 (0,65–0,78)	*	0,59 (0,52–0,66)	
**CST5**	5,64 (5,36–5,92)	**	5,81 (5,66–5,95)		5,77 (5,64–5,91)	*
**DNER**	7,27 (6,99–7,55)	**	8,10 (7,82–8,38)	*	7,92 (7,66–8,17)	**
**FGF-5**	1,94 (1,62–2,26)	**	2,88 (2,57–3,19)		2,66 (2,35–2,96)	**
**Flt3L**	7,45 (7,16–7,74)	**	7,95 (7,65–8,26)		8,00 (7,7–8,31)	**
**IL-18R1**	2,16 (1,74–2,58)		2,52 (2,09–2,94)		2,33 (1,93–2,73)	
**LIF-R**	2,13 (1,93–2,33)	**	2,50 (2,29–2,71)		2,42 (2,27–2,56)	**
**OSM**	4,04 (3,41–4,68)	*	4,85 (4,14–5,56)		4,87 (4,32–5,43)	*

Comparison of protein expression levels (mean NPX ± 0.95 CI within parentheses) between time points for each protein. Repeated measures ANOVA and Fisher LSD test for post-hoc analyses was used for comparisons when data were normally distributed and assumption of compound symmetry was met. Nonparametric equivalents Friedman’s ANOVA and Wilcoxon test were used when parametric statistics were deemed inappropriate; asterisks indicate level of significance for each comparison (* = p<0,05;

** = p<0,005).

Fourteen of the 30 proteins in Group C (Table 5) showed statistically significant late peak defined as significantly higher day 10 vs day 4 values, i.e. 4E-BP1, ADA, CASP-8, CCL19, CCL23 (MIP-3), CD40, CDCP1, CXCL1, CXCL6, CXCL8 (IL-8), CXCL9, IL-18, SIRT2 and uPA. Ten of them also showed statistically significant increasing trend during the entire observation period, that is significantly higher day 10 vs day 4 *and* day 4 vs day 1 values (i.e. 4E-BP1, ADA, CCL19, CCL23, CD40, CDCP1, CXCL1, CXCL6, CXCL9 and uPA).

**Table 5:** Group C proteins.

	DAY 1	1v4	DAY 4	4v10	DAY 10	1v10
**4E-BP1**	1,45 (1,07–1,83)	**	4,04 (3,10–4,97)	**	6,18 (5,49–6,86)	**
**ADA**	4,02 (3,71–4,33)	**	5,30 (4,73–5,87)	*	5,86 (5,35–6,36)	**
**CASP-8**	1,45 (1,13–1,78)		1,73 (1,33–2,14)	**	2,51 (2,09–2,93)	**
**CCL13 (MCP-4)**	0,75 (0,54–0,95)		0,66 (0,57–0,76)		1,14 (0,9–1,39)	
**CCL19**	7,74 (7,27–8,21)	**	10,38 (9,66–11,1)	*	11,08 (10,44–11,72)	**
**CCL23 (MIP-3)**	3,86 (3,38–4,34)	**	5,89 (5,27–6,51)	**	7,26 (6,65–7,86)	**
**CCL7 (MCP-3)**	5,29 (4,65–5,93)	*	6,27 (5,60–6,94)		6,65 (5,92–7,39)	**
**CCL8 (MCP-2)**	6,98 (6,34–7,62)	*	8,13 (7,42–8,84)		8,88 (8,24–9,52)	**
**CD40**	6,12 (5,75–6,49)	**	6,87 (6,39–7,35)	**	7,82 (7,33–8,30)	**
**CD5**	1,29 (0,92–1,67)	*	1,73 (1,42–2,04)		1,88 (1,50–2,27)	*
**CDCP-1**	3,71 (3,48–3,95)	**	4,37 (4,11–4,63)	**	4,74 (4,42–5,07)	**
**CXCL1**	7,44 (6,81–8,06)	*	8,80 (7,98–9,62)	*	9,55 (8,79–10,32)	**
**CXCL10**	7,77 (7,04–8,50)	**	10,44 (9,67–11,22)		10,92 (10,33–11,52)	**
**CXCL11**	2,84 (2,41–3,28)	**	3,98 (3,40–4,56)		4,30 (3,83–4,76)	**
**CXCL6**	4,41 (3,74–5,07)	**	6,81 (5,85–7,77)	**	8,57 (7,68–9,45)	**
**CXCL8 (IL-8)**	12,80 (12,3–13,29)		12,89 (12,25–13,52)	*	13,34 (12,73–13,95)	
**CXCL9**	3,38 (2,96–3,80)	**	4,68 (4,15–5,22)	**	5,86 (5,2–6,52)	**
**EN-RAGE**	1,99 (1,46–2,52)	**	3,71 (3,10–4,32)		4,12 (3,49–4,75)	**
**HGF**	5,40 (4,97–5,83)	**	6,44 (6,06–6,81)		6,76 (6,38–7,15)	**
**IL-10RB**	2,06 (1,74–2,39)	**	2,65 (2,31–3)		2,88 (2,53–3,24)	**
**IL-18**	2,88 (2,35–3,41)		3,08 (2,43–3,74)	**	4,18 (3,64–4,73)	**
**IL-6**	9,55 (8,92–10,18)		10,45 (9,51–11,4)		10,79 (10,01–11,57)	*
**IL-7**	1,22 (1,14–1,31)	*	1,48 (1,32–1,63)		1,57 (1,43–1,71)	**
**OPG**	9,38 (8,78–9,98)		9,79 (9,23–10,36)		10,32 (9,74–10,9)	
**SIRT-2**	2,56 (2,09–3,03)		2,96 (2,46–3,6)	*	3,49 (2,97–4,02)	**
**STAMPB**	1,90 (1,58–2,22)		2,12 (1,73–2,52)		2,54 (2,15–2,93)	**
**TGF-β1**	3,95 (3,58–4,32)	*	4,57 (4,11–5,02)		4,80 (4,32–5,27)	**
**TNF-β**	1,03 (0,74–1,31)		0,81 (0,67–0,95)		1,27 (1,02–1,51)	
**TWEAK**	7,51 (7,21–7,81)	**	8,22 (7,9–8,55)		8,40 (8,11–8,69)	**
**uPA**	7,34 (7,01–7,66)	**	8,60 (8,17–9,04)	**	10,02 (9,47–10,57)	**

Comparison of protein expression levels (mean NPX ± 0.95 CI within parentheses) between time points for each protein. Repeated measures ANOVA and Fisher LSD test for post-hoc analyses was used for comparisons when data were normally distributed and assumption of compound symmetry was met. Nonparametric equivalents Friedman’s ANOVA and Wilcoxon test were used when parametric statistics were deemed inappropriate; asterisks indicate level of significance for each comparison (* = p<0,05;

** = p<0,005).

Four of the Group D proteins (Table 6) showed biphasic peaks at day 1 and day 10, i.e. CCL 25, CD6, MMP1 and VEGF-A, but only VEGF-A had a statistically significant difference between day 1 vs day 4 values. The remaining 9 proteins CCL2 (MCP-1), CSF-1, CX3CL1, CXCL5, IL-10, IL-12RB, SCF, TNFRSF9 and TNFSF14 showed fairly stable values throughout the observation period.

**Table 6:** Group D proteins.

	DAY 1	1v4	DAY 4	4v10	DAY 10	1v10
**CCL2 (MCP-1)**	13,15 (13,04–13,26)		13,04 (12,86–13,23)		13,02 (12,85–13,18)	
**CCL25**	1,68 (1,34–2,03)		1,34 (1,16–1,52)		1,56 (1,28–1,85)	
**CD6**	0,69 (0,36–1,02)		0,49 (0,36–0,62)		0,60 (0,45–0,75)	
**CSF-1**	6,30 (5,92–6,68)		6,54 (6,18–6,90)		6,29 (6,01–6,58)	
**CX3CL1**	2,98 (2,67–3,29)		3,31 (2,96–3,67)		3,45 (3,14–3,77)	
**CXCL5**	8,35 (7,59–9,11)		8,52 (7,81–9,23)		8,59 (7,89–9,30)	
**IL-10**	2,63 (2,18–3,07)		2,88 (2,19–3,56)		2,54 (2,06–3,02)	
**IL-12B**	1,64 (1,27–2,01)		1,77 (0,66–2,88)		1,37 (1,25–1,49)	
**MMP1**	0,94 (0,61–1,26)		0,76 (0,15–1,36)		0,83 (0,42–1,25)	
**SCF**	3,08 (2,64–3,51)		3,39 (3,01–3,78)		3,52 (3,09–3,95)	
**TNFRSF9**	2,63 (2,27–3)		2,96 (2,55–3,38)		2,86 (2,48–3,25)	
**TNFSF14**	0,90 (0,49–1,31)		0,72 (0,3–1,13)		0,72 (0,33–1,11)	
**VEGF-A**	9,93 (9,57–10,29)	*	9,51 (9,14–9,87)		9,79 (9,44–10,13)	

Comparison of protein expression levels (mean NPX ± 0.95 CI within parentheses) between time points for each protein. Repeated measures ANOVA and Fisher LSD test for post-hoc analyses was used for comparisons when data were normally distributed and assumption of compound symmetry was met. Nonparametric equivalents Friedman’s ANOVA and Wilcoxon test were used when parametric statistics were deemed inappropriate; asterisks indicate level of significance for each comparison (* = p<0,05;

** = p<0,005).

## Discussion

In this study we present a concise data set of the sequential production of multiple inflammation-related proteins in the CSF of patients during the first 10 days post SAH. This is the first report of Proximity Extension Assay (PEA) technology being used for measuring complex protein expression levels in the context of SAH. Similar studies were recently published in patients with traumatic brain injury (TBI) and trigeminal neuralgia, as well as numerous other non-neurosurgical conditions (such as neuropathic pain, cardiovascular diseases, gastric cancer, etc.) [29–31].

The analyses in the present study were performed in CSF compartment alone (not plasma or cerebral interstitial fluid) as this seems more suitable to describe the disease pathophysiology, given also that major early and late clinical complications (i.e. CV and chronic hydrocephalus) spatially correlate best with this compartment. Many of the included proteins have previously been associated to SAH inflammation, for example IL-1ra, IL-6, IL-8, TNF-a, LIF, MCP-1, and VEGF-A [32]. On the other hand, there is scarce or non-existing literature on many other proteins, some of which showed interesting temporal patterns and statistically significant peaks and trends in the present study, such as LIF, CCL11, CCL28, 4E-BP1, CD40, CXCL6, CXCL9, and IL-18 [20, 33–36].

### Group A

Eleven proteins showed higher day 1 values and decreasing trends throughout the observation period (Tables 2 and 3). Four of them (CCL11, CCL4, LIF and TGF-α) showed statistically significant early peaks with day 1 > day 4 levels but no statistically significant decreasing trends were observed. CCL4 (also known as MIP-1β) and CCL11 are members of the chemokine family (C-C motif) and are involved in chemotaxis of macrophages and activated T-cells, respectively, as well as other proinflammatory actions. Their early peak can possibly be associated to the recruitment of leukocytes at the site of the bleeding. LIF is a cytokine involved in activation of signaling pathways that regulate cell growth among other actions. A similar early peak in serum has been observed previously [37]. TGF-α has not been studied in the SAH literature.

### Group B

Among the 10 proteins included in this group only two (CCL28 and DNER) showed statistically significant middle peaks, that is significantly higher day 4 values than *both* day 1 *and* day 10 (Tables 2 and 4). Production of the chemokine CCL28 is induced by other proinflammatory cytokines and its chemotactic actions are exerted on B- and T-cells and eosinophils. Delta and Notch-like Epidermal growth factor-Receptor (DNER) is an activator of NOTCH1 pathway. None of these proteins have been studied in a SAH context earlier and their potential involvement in the SAH complications, mainly CV that coincides temporally with the observed middle peaks, should be examined.

### Group C

Thirty proteins showed higher levels towards the end of the observation period, reflecting a more delayed activation post SAH that may indicate an involvement in the healing processes or the development of late complications, such as late vasospasm, posthemorrhagic hydrocephalus, etc. (Tables 2 and 5). Fourteen proteins showed statistically significant late peaks, meaning significantly higher day 10 than day 4 values; ten of them even showed significantly increasing trends throughout the observation period with day 1 < day 4 < day 10 values.

Signaling pathway molecule 4E-BP1 has been implicated in the development of vasospasm in a canine SAH-model but no reports on human studies are available [35]. Chemokines CCL19, CCL23, CCL8 and CXCL6, all potent chemotactic agents for a variety of immune cells such as lymphocytes, resting T-cells/monocytes and neutrophil granulocytes, have been studied in other CNS-related inflammatory diseases but not in SAH patients earlier [38]. Chemokines CXCL1, CXCL9, CXCL10 and CXCL11 demonstrated a similar pattern of late activation alongside with monocyte and T-cells infiltration within the CNS and were associated with DCI occurrence in a recent study by Mohme et al. [39]. Protein CD40, a member of TNF family, is found on antigen-presenting cells and mediates multiple inflammatory responses. Elevated serum levels of CD40 have been associated with poor outcome and severity of neurological deficits in SAH patients [36, 40]. IL-18, a pro-inflammatory cytokine involved in the synthesis of inflammatory mediators, has recently been shown to be a predictor of early brain injury and clinical prognosis in SAH patients as elevated concentrations correlated to cerebral edema and acute hydrocephalus [20]. It should be noted though that the observed temporal pattern of IL-18 in that study differed from our study as we demonstrated a late peak of this cytokine. Urokinase (or uPA) is a serum protease that activates plasminogen to plasmin which in turn leads to thrombolysis and tissue degradation. Plasma concentrations of its receptor (soluble uPA-receptor) was shown not to correlate with neurological outcome post SAH [41].

### Group D

VEGF-A is a well-studied biomarker in SAH [42–44]. Upon binding to its receptors it promotes endothelial cell proliferation and cell migration and increases permeability of the blood vessels. It has been described to play an important role in the blood brain barrier integrity and development of brain edema and its suppression has reportedly ameliorated EBI in experimental SAH [45]. Higher levels on admission have also been correlated with worse outcomes in patients [46]. The early peak noticed in our study can be explained by these actions that usually take place early post SAH. The late peak is less clear (nor statistically significant) but may indicate involvement in the development of chronic hydrocephalus as an experimental study suggested previously [47].

CCL2 or MCP-1 is a strong chemotactic agent for monocytes and has been correlated with bleeding severity and development of DCI [19, 34, 48]. Higher serum concentrations of MCP-1 correlated with poor outcome and higher CSF concentrations with angiographically demonstrated vasospasm in an early study by Kim et al. [49]. Of note is the high NPX values of this biomarker that were among the highest observed values in our material. No peak or trend was observed in our study but en ultra-early peak before the first sample and a strong activation throughout the observation period cannot be ruled out.

The absence of a specific pattern of activation for the remaining Group D proteins may be attributed to the fact that these proteins are only relevant for a very short period of time that was not captured in the time points of the present analysis or not relevant at all.

### General considerations

The great variability of the expression levels and temporal patterns of the measured inflammation-related proteins is an indicator of the complexity of the inflammatory response after SAH. Many of the included proteins are well established biomarkers in the SAH research both in humans and in preclinical animal models while others are novel in the SAH field. Their exact role as well as interplay with each other is not easy to establish, especially considering the fact that many of these substances are described to play both a detrimental and a beneficial role in the disease course depending on the time after bleeding [50]. Bioinformatics analyses could address this complex interplay between and within groups of proteins with similar features (such as same family or similar temporal pattern of activation) in order to better elucidate their role in the SAH pathophysiology in studies specifically designed for that purpose. The current study provides a rough description of the inflammatory profile of the acute phase of SAH and may serve as a pilot for the design of such biomarker studies.

### Limitations

A limitation with the study is that the PEA analysis method in its presently available form does not give absolute protein concentrations. However, the protein expression levels illustrate the relative concentrations and how these change over time. They also illustrate the temporal relations between the levels of the different proteins. The study may be limited by the relatively small number of patients included. Another drawback may be the fact that the study is limited to the CSF compartment; similar analyses could be performed in the cerebral interstitial fluid and plasma, giving the opportunity to compare protein levels in the different fluid compartments providing a more thorough inflammatory profile of the acute phase of the disease. Comparisons with healthy individuals could also serve as an indicator of the intensity of activation for each protein. Moreover, correlations of the protein expression levels with clinical parameters were not performed in this study, nor were bioinformatics analyses, as the main goal was to provide general information of as many proteins as possible in order to look for patterns of expression for further investigation. Finally, future SAH studies with a larger number of time points are warranted to describe the temporal pattern of each protein more accurately.

## Conclusion

The temporal patterns of expression of multiple inflammation-related proteins in the acute phase of SAH are reported in this explorative study providing an inflammatory profile of the disease that facilitates further research in the field of protein biomarkers. Proximity Extension Assay technology enables the measurement of the expression levels of several proteins simultaneously in small amounts of sample with high specificity, adding a useful tool in the quest of finding relevant biomarkers to better describe and understand the complex pathophysiology of SAH.

## Supporting information

S1 Data (XLSX)

## List of abbreviations

BBB: blood-brain barrier
CBF: cerebral blood flow
CSD: cortical spreading depolarization
CSF: cerebrospinal fluid
CV: cerebral vasospasm
DCI: delayed cerebral ischemia
SAH: subarachnoid hemorrhage
PEA: Proximity Extension Assay
EBI: early brain injury
EVD: external ventricular drainage
GOS: Glasgow outcome scale
ICP: intracranial pressure
LOD: limit of detection
MD: microdialysis
NPX: normalized protein expression
TBI: traumatic brain injury
TNF: tumor necrosis factor
WFNS: World Federation of Neurosurgical Societies

## References

[1] Macdonald RL, Schweizer TA. Spontaneous subarachnoid haemorrhage. Lancet (London, England). 2017;389(10069):655–66. Epub 2016/09/18. doi: 10.1016/S0140-6736(16)30668-7 . 27637674

[2] Budohoski KP, Guilfoyle M, Helmy A, Huuskonen T, Czosnyka M, Kirollos R, et al. The pathophysiology and treatment of delayed cerebral ischaemia following subarachnoid haemorrhage. Journal of neurology, neurosurgery, and psychiatry. 2014;85(12):1343–53. Epub 2014/05/23. doi: 10.1136/jnnp-2014-307711 . 24847164

[3] Ohkuma H, Manabe H, Tanaka M, Suzuki S. Impact of cerebral microcirculatory changes on cerebral blood flow during cerebral vasospasm after aneurysmal subarachnoid hemorrhage. Stroke. 2000;31(7):1621–7. Epub 2000/07/08. doi: 10.1161/01.str.31.7.1621 . 10884463

[4] Alaraj A, Charbel FT, Amin-Hanjani S. Peri-operative measures for treatment and prevention of cerebral vasospasm following subarachnoid hemorrhage. Neurological research. 2009;31(6):651–9. Epub 2009/01/10. doi: 10.1179/174313209X382395 . 19133166

[5] Macdonald RL, Kassell NF, Mayer S, Ruefenacht D, Schmiedek P, Weidauer S, et al. Clazosentan to overcome neurological ischemia and infarction occurring after subarachnoid hemorrhage (CONSCIOUS-1): randomized, double-blind, placebo-controlled phase 2 dose-finding trial. Stroke. 2008;39(11):3015–21. Epub 2008/08/09. doi: 10.1161/STROKEAHA.108.519942 . 18688013

[6] Macdonald RL, Higashida RT, Keller E, Mayer SA, Molyneux A, Raabe A, et al. Clazosentan, an endothelin receptor antagonist, in patients with aneurysmal subarachnoid haemorrhage undergoing surgical clipping: a randomised, double-blind, placebo-controlled phase 3 trial (CONSCIOUS-2). The Lancet Neurology. 2011;10(7):618–25. Epub 2011/06/07. doi: 10.1016/S1474-4422(11)70108-9 . 21640651

[7] Schneider UC, Xu R, Vajkoczy P. Inflammatory events following subarachnoid hemorrhage (SAH). Current neuropharmacology. 2018. Epub 2018/04/14. doi: 10.2174/1570159x16666180412110919 . 29651951 PMC6251050

[8] Pluta RM, Hansen-Schwartz J, Dreier J, Vajkoczy P, Macdonald RL, Nishizawa S, et al. Cerebral vasospasm following subarachnoid hemorrhage: time for a new world of thought. Neurological research. 2009;31(2):151–8. Epub 2009/03/21. doi: 10.1179/174313209X393564 ; PubMed Central PMCID: PMC2706525. 19298755 PMC2706525

[9] Sehba FA, Pluta RM, Zhang JH. Metamorphosis of subarachnoid hemorrhage research: from delayed vasospasm to early brain injury. Molecular neurobiology. 2011;43(1):27–40. Epub 2010/12/17. doi: 10.1007/s12035-010-8155-z ; PubMed Central PMCID: PMC3023855. 21161614 PMC3023855

[10] Macdonald RL. Delayed neurological deterioration after subarachnoid haemorrhage. Nature reviews Neurology. 2014;10(1):44–58. Epub 2013/12/11. doi: 10.1038/nrneurol.2013.246 . 24323051

[11] Lucke-Wold BP, Logsdon AF, Manoranjan B, Turner RC, McConnell E, Vates GE, et al. Aneurysmal Subarachnoid Hemorrhage and Neuroinflammation: A Comprehensive Review. International journal of molecular sciences. 2016;17(4):497. Epub 2016/04/07. doi: 10.3390/ijms17040497 ; PubMed Central PMCID: PMC4848953. 27049383 PMC4848953

[12] Provencio JJ. Inflammation in subarachnoid hemorrhage and delayed deterioration associated with vasospasm: a review. Acta neurochirurgica Supplement. 2013;115:233–8. Epub 2012/08/15. doi: 10.1007/978-3-7091-1192-5_42 ; PubMed Central PMCID: PMC3597075. 22890674 PMC3597075

[13] de Oliveira Manoel AL, Macdonald RL. Neuroinflammation as a Target for Intervention in Subarachnoid Hemorrhage. Frontiers in neurology. 2018;9:292. Epub 2018/05/18. doi: 10.3389/fneur.2018.00292 ; PubMed Central PMCID: PMC5941982. 29770118 PMC5941982

[14] Mathiesen T, Edner G, Ulfarsson E, Andersson B. Cerebrospinal fluid interleukin-1 receptor antagonist and tumor necrosis factor-alpha following subarachnoid hemorrhage. Journal of neurosurgery. 1997;87(2):215–20. Epub 1997/08/01. doi: 10.3171/jns.1997.87.2.0215 9254084

[15] Fassbender K, Hodapp B, Rossol S, Bertsch T, Schmeck J, Schutt S, et al. Inflammatory cytokines in subarachnoid haemorrhage: association with abnormal blood flow velocities in basal cerebral arteries. Journal of neurology, neurosurgery, and psychiatry. 2001;70(4):534–7. Epub 2001/03/20. doi: 10.1136/jnnp.70.4.534 ; PubMed Central PMCID: PMC1737308. 11254783 PMC1737308

[16] Schoch B, Regel JP, Wichert M, Gasser T, Volbracht L, Stolke D. Analysis of intrathecal interleukin-6 as a potential predictive factor for vasospasm in subarachnoid hemorrhage. Neurosurgery. 2007;60(5):828–36; discussion -36. Epub 2007/04/27. doi: 10.1227/01.NEU.0000255440.21495.80 . 17460517

[17] Sarrafzadeh A, Schlenk F, Gericke C, Vajkoczy P. Relevance of cerebral interleukin-6 after aneurysmal subarachnoid hemorrhage. Neurocritical care. 2010;13(3):339–46. Epub 2010/08/21. doi: 10.1007/s12028-010-9432-4 . 20725805

[18] Helbok R, Schiefecker AJ, Beer R, Dietmann A, Antunes AP, Sohm F, et al. Early brain injury after aneurysmal subarachnoid hemorrhage: a multimodal neuromonitoring study. Critical care (London, England). 2015;19:75. Epub 2015/04/19. doi: 10.1186/s13054-015-0809-9 ; PubMed Central PMCID: PMC4384312. 25887441 PMC4384312

[19] Niwa A, Osuka K, Nakura T, Matsuo N, Watabe T, Takayasu M. Interleukin-6, MCP-1, IP-10, and MIG are sequentially expressed in cerebrospinal fluid after subarachnoid hemorrhage. Journal of neuroinflammation. 2016;13(1):217. Epub 2016/09/01. doi: 10.1186/s12974-016-0675-7 ; PubMed Central PMCID: PMC5006407. 27576738 PMC5006407

[20] Lv SY, Wu Q, Liu JP, Shao J, Wen LL, Xue J, et al. Levels of Interleukin-1beta, Interleukin-18, and Tumor Necrosis Factor-alpha in Cerebrospinal Fluid of Aneurysmal Subarachnoid Hemorrhage Patients May Be Predictors of Early Brain Injury and Clinical Prognosis. World neurosurgery. 2018;111:e362–e73. Epub 2017/12/27. doi: 10.1016/j.wneu.2017.12.076 . 29277532

[21] Wang Y, Zhong M, Tan XX, Yang YJ, Chen WJ, Liu W, et al. Expression change of interleukin-8 gene in rabbit basilar artery after subarachnoid hemorrhage. Neuroscience bulletin. 2007;23(3):151–5. Epub 2007/07/07. doi: 10.1007/s12264-007-0022-1 ; PubMed Central PMCID: PMC5550629. 17612593 PMC5550629

[22] Hanafy KA. The role of microglia and the TLR4 pathway in neuronal apoptosis and vasospasm after subarachnoid hemorrhage. Journal of neuroinflammation. 2013;10:83. Epub 2013/07/16. doi: 10.1186/1742-2094-10-83 ; PubMed Central PMCID: PMC3750560. 23849248 PMC3750560

[23] Assarsson E, Lundberg M, Holmquist G, Bjorkesten J, Thorsen SB, Ekman D, et al. Homogenous 96-plex PEA immunoassay exhibiting high sensitivity, specificity, and excellent scalability. PloS one. 2014;9(4):e95192. Epub 2014/04/24. doi: 10.1371/journal.pone.0095192 ; PubMed Central PMCID: PMC3995906. 24755770 PMC3995906

[24] Greenwood C, Ruff D, Kirvell S, Johnson G, Dhillon HS, Bustin SA. Proximity assays for sensitive quantification of proteins. Biomolecular detection and quantification. 2015;4:10–6. Epub 2016/04/15. doi: 10.1016/j.bdq.2015.04.002 ; PubMed Central PMCID: PMC4822221. 27077033 PMC4822221

[25] Fisher CM, Kistler JP, Davis JM. Relation of cerebral vasospasm to subarachnoid hemorrhage visualized by computerized tomographic scanning. Neurosurgery. 1980;6(1):1–9. Epub 1980/01/01. doi: 10.1227/00006123-198001000-00001 . 7354892

[26] Teasdale GM, Drake CG, Hunt W, Kassell N, Sano K, Pertuiset B, et al. A universal subarachnoid hemorrhage scale: report of a committee of the World Federation of Neurosurgical Societies. Journal of neurology, neurosurgery, and psychiatry. 1988;51(11):1457. Epub 1988/11/01. doi: 10.1136/jnnp.51.11.1457 ; PubMed Central PMCID: PMC1032822. 3236024 PMC1032822

[27] Johansson M, Cesarini KG, Contant CF, Persson L, Enblad P. Changes in intervention and outcome in elderly patients with subarachnoid hemorrhage. Stroke. 2001;32(12):2845–949. Epub 2001/12/12. doi: 10.1161/hs1201.099416 . 11739985

[28] Jennett B, Bond M. Assessment of outcome after severe brain damage. Lancet (London, England). 1975;1(7905):480–4. Epub 1975/03/01. doi: 10.1016/s0140-6736(75)92830-5 46957

[29] Dyhrfort P, Shen Q, Clausen F, Thulin M, Enblad P, Kamali-Moghaddam M, et al. Monitoring of Protein Biomarkers of Inflammation in Human Traumatic Brain Injury Using Microdialysis and Proximity Extension Assay Technology in Neurointensive Care. Journal of neurotrauma. 2019;36(20):2872–85. Epub 2019/04/25. doi: 10.1089/neu.2018.6320 ; PubMed Central PMCID: PMC6761596. 31017044 PMC6761596

[30] Ericson H, Abu Hamdeh S, Freyhult E, Stiger F, Backryd E, Svenningsson A, et al. Cerebrospinal fluid biomarkers of inflammation in trigeminal neuralgia patients operated with microvascular decompression. Pain. 2019;160(11):2603–11. Epub 2019/08/03. doi: 10.1097/j.pain.0000000000001649 . 31373951

[31] Backryd E, Lind AL, Thulin M, Larsson A, Gerdle B, Gordh T. High levels of cerebrospinal fluid chemokines point to the presence of neuroinflammation in peripheral neuropathic pain: a cross-sectional study of 2 cohorts of patients compared with healthy controls. Pain. 2017;158(12):2487–95. Epub 2017/09/21. doi: 10.1097/j.pain.0000000000001061 ; PubMed Central PMCID: PMC5690569. 28930774 PMC5690569

[32] Zeiler FA, Thelin EP, Czosnyka M, Hutchinson PJ, Menon DK, Helmy A. Cerebrospinal Fluid and Microdialysis Cytokines in Aneurysmal Subarachnoid Hemorrhage: A Scoping Systematic Review. Frontiers in neurology. 2017;8:379. Epub 2017/08/30. doi: 10.3389/fneur.2017.00379 ; PubMed Central PMCID: PMC5550693. 28848487 PMC5550693

[33] Hollig A, Remmel D, Stoffel-Wagner B, Schubert GA, Coburn M, Clusmann H. Association of early inflammatory parameters after subarachnoid hemorrhage with functional outcome: A prospective cohort study. Clinical neurology and neurosurgery. 2015;138:177–83. Epub 2015/09/12. doi: 10.1016/j.clineuro.2015.08.030 . 26355810

[34] Savarraj JPJ, Parsha K, Hergenroeder GW, Zhu L, Bajgur SS, Ahn S, et al. Systematic model of peripheral inflammation after subarachnoid hemorrhage. Neurology. 2017;88(16):1535–45. Epub 2017/03/21. doi: 10.1212/WNL.0000000000003842 ; PubMed Central PMCID: PMC5395070. 28314864 PMC5395070

[35] Zhang W, Khatibi NH, Yamaguchi-Okada M, Yan J, Chen C, Hu Q, et al. Mammalian target of rapamycin (mTOR) inhibition reduces cerebral vasospasm following a subarachnoid hemorrhage injury in canines. Experimental neurology. 2012;233(2):799–806. Epub 2011/12/20. doi: 10.1016/j.expneurol.2011.11.046 . 22177999

[36] Kubo Y, Koji T, Yoshida J, Ogawa A, Ogasawara K. Predicting neurological deficit severity due to subarachnoid haemorrhage: soluble CD40 ligand and platelet-derived growth factor-BB. Critical care and resuscitation: journal of the Australasian Academy of Critical Care Medicine. 2016;18(4):242–6. Epub 2016/12/03. . 27903205

[37] Hollig A, Stoffel-Wagner B, Clusmann H, Veldeman M, Schubert GA, Coburn M. Time Courses of Inflammatory Markers after Aneurysmal Subarachnoid Hemorrhage and Their Possible Relevance for Future Studies. Frontiers in neurology. 2017;8:694. Epub 2018/01/10. doi: 10.3389/fneur.2017.00694 ; PubMed Central PMCID: PMC5744005. 29312122 PMC5744005

[38] Lepennetier G, Hracsko Z, Unger M, Van Griensven M, Grummel V, Krumbholz M, et al. Cytokine and immune cell profiling in the cerebrospinal fluid of patients with neuro-inflammatory diseases. Journal of neuroinflammation. 2019;16(1):219. Epub 2019/11/16. doi: 10.1186/s12974-019-1601-6 ; PubMed Central PMCID: PMC6857241. 31727097 PMC6857241

[39] Mohme M, Sauvigny T, Mader MM, Schweingruber N, Maire CL, Runger A, et al. Immune Characterization in Aneurysmal Subarachnoid Hemorrhage Reveals Distinct Monocytic Activation and Chemokine Patterns. Translational stroke research. 2019. Epub 2019/12/21. doi: 10.1007/s12975-019-00764-1 . 31858408

[40] Chen XD, Sun J, Lu C, Ba HJ, Chen MH, Lin JH, et al. The prognostic value of plasma soluble CD40 ligand levels following aneurysmal subarachnoid hemorrhage. Thrombosis research. 2015;136(1):24–9. Epub 2015/05/07. doi: 10.1016/j.thromres.2015.03.025 . 25944664

[41] Kiiski H, Jalkanen V, Ala-Peijari M, Hamalainen M, Moilanen E, Peltola J, et al. Plasma Soluble Urokinase-Type Plasminogen Activator Receptor Is Not Associated with Neurological Outcome in Patients with Aneurysmal Subarachnoid Hemorrhage. Frontiers in neurology. 2017;8:144. Epub 2017/05/02. doi: 10.3389/fneur.2017.00144 ; PubMed Central PMCID: PMC5394110. 28458650 PMC5394110

[42] Kusaka G, Ishikawa M, Nanda A, Granger DN, Zhang JH. Signaling pathways for early brain injury after subarachnoid hemorrhage. Journal of cerebral blood flow and metabolism: official journal of the International Society of Cerebral Blood Flow and Metabolism. 2004;24(8):916–25. Epub 2004/09/15. doi: 10.1097/01.WCB.0000125886.48838.7E . 15362722

[43] Mellergard P, Sjogren F, Hillman J. Release of VEGF and FGF in the extracellular space following severe subarachnoidal haemorrhage or traumatic head injury in humans. British journal of neurosurgery. 2010;24(3):261–7. Epub 2010/05/15. doi: 10.3109/02688690903521605 . 20465454

[44] Wiciński M, Al Drawi AS, Malinowski B, Stolarek W. Evaluation of Vascular Endothelial Growth Factor A and Selected Parameters of Coagulation and Fibrinolysis in a Group of Patients with Subarachnoid Haemorrhage. BioMed research international. 2019;2019:8759231. Epub 2019/07/31. doi: 10.1155/2019/8759231 ; PubMed Central PMCID: PMC6644279. 31360727 PMC6644279

[45] Liu L, Fujimoto M, Kawakita F, Nakano F, Imanaka-Yoshida K, Yoshida T, et al. Anti-Vascular Endothelial Growth Factor Treatment Suppresses Early Brain Injury After Subarachnoid Hemorrhage in Mice. Molecular neurobiology. 2016;53(7):4529–38. Epub 2015/08/21. doi: 10.1007/s12035-015-9386-9 . 26289408

[46] Gris T, Laplante P, Thebault P, Cayrol R, Najjar A, Joannette-Pilon B, et al. Innate immunity activation in the early brain injury period following subarachnoid hemorrhage. Journal of neuroinflammation. 2019;16(1):253. Epub 2019/12/06. doi: 10.1186/s12974-019-1629-7 ; PubMed Central PMCID: PMC6894125. 31801576 PMC6894125

[47] Chu SH, Feng DF, Ma YB, Zhang H, Zhu ZA, Li ZQ, et al. Expression of HGF and VEGF in the cerebral tissue of adult rats with chronic hydrocephalus after subarachnoid hemorrhage. Molecular medicine reports. 2011;4(5):785–91. Epub 2011/06/07. doi: 10.3892/mmr.2011.500 . 21643626

[48] Ahn SH, Savarraj JPJ, Parsha K, Hergenroeder GW, Chang TR, Kim DH, et al. Inflammation in delayed ischemia and functional outcomes after subarachnoid hemorrhage. Journal of neuroinflammation. 2019;16(1):213. Epub 2019/11/13. doi: 10.1186/s12974-019-1578-1 ; PubMed Central PMCID: PMC6849179. 31711504 PMC6849179

[49] Kim GH, Kellner CP, Hahn DK, Desantis BM, Musabbir M, Starke RM, et al. Monocyte chemoattractant protein-1 predicts outcome and vasospasm following aneurysmal subarachnoid hemorrhage. Journal of neurosurgery. 2008;109(1):38–43. Epub 2008/07/03. doi: 10.3171/JNS/2008/109/7/0038 . 18593272

[50] Gadani SP, Walsh JT, Lukens JR, Kipnis J. Dealing with Danger in the CNS: The Response of the Immune System to Injury. Neuron. 2015;87(1):47–62. Epub 2015/07/04. doi: 10.1016/j.neuron.2015.05.019 ; PubMed Central PMCID: PMC4491143. 26139369 PMC4491143

